# Association of homocysteine with type 2 diabetes: a meta-analysis implementing Mendelian randomization approach

**DOI:** 10.1186/1471-2164-14-867

**Published:** 2013-12-10

**Authors:** Tao Huang, JingJing Ren, Jinyan Huang, Duo Li

**Affiliations:** 1 Department of International Health, Johns Hopkins Bloomberg School of Public Health, Baltimore, MD 21205, USA; 2 Department of Food Science and Nutrition, Zhejiang University, 268 Kaixuan Road, Hangzhou 310029, China; 3 APCNS Centre of Nutrition and Food Safety, Hangzhou, China; 4 The First Affiliated Hospital, Medical College, Zhejiang University, Hangzhou, China; 5 Department of Biostatistics, Harvard School of Public Health, Boston, MA 02115, USA

**Keywords:** Homocysteine, MTHFR, Type 2 diabetes

## Abstract

**Background:**

We tested the hypothesis that elevated homocysteine (Hcy) level is causally associated with increased risk of type 2 diabetes mellitus (T2DM).

**Results:**

The meta-analysis and Mendelian randomization analysis were performed among 4011 cases and 4303 controls. The absolute pooled mean Hcy concentration in subjects with *MTHFR* 677TT was 5.55 μmol/L (95% CI, 1.33 to 9.77) greater than that in subjects with *MTHFR* 677CC in T2DM. Overall, the T allele of the *MTHFR* 677 C > T conferred a greater risk for T2DM [Random effect (RE) OR = 1.31(1.17-1.64), I^2^ = 41.0%, p = 0.055]. The random effect (RE) pooled OR associated with T2DM for *MTHFR* 677TT relative to the 677CC was [RE OR = 1.38(1.18-1.62)]. The fixed-effect pooled OR of the association for the *MTHFR* 677 TT vs CT was 1.29 (95% CI, 1.09-1.51). *MTHFR* 677 TT showed a significantly higher risk for T2DM compared with *MTHFR* 677 CC + CT [Fixed effect (FE) OR = 1.32(1.14-1.54), I^2^ = 0.0%, p = 0.686]. The absolute pooled mean Hcy concentration in individuals with T2DM was 0.94 μmol/L (95% CI, 0.40-1.48) greater than that in control subjects. The estimated causal OR associated with T2DM was 1.29 for 5 μmol/L increment in Hcy.

**Conclusions:**

Our findings provided strong evidence on the causal association of Hcy level with the development of T2DM.

## Background

The pathophysiology of type 2 diabetes mellitus (T2DM) characterized by a high incidence of vascular complications is complex and multifactorial [1]. The major cause of T2DM and its complications has not yet been clarified. In recent years, plasma homocysteine (Hcy) level, a sulphur-containing non-protein amino acid in the metabolism of methionine, has been reported to be associated with the vascular complications of diabetes [2]. In patients with diabetes, elevated Hcy levels were associated with insulin resistance [3] and nephropathy [4]. Of note, several studies have demonstrated that elevated Hcy levels predict the risk of death or coronary events in patients with T2DM [5,6]. However, conflicting results regarding the Hcy level in patients with diabetes have been reported. Some studies found that plasma Hcy levels were increased [7,8], unchanged [9-11], or decreased [12,13] in patients with T2DM.

The candidate gene approach is widely used for identifying genes involved in complex human diseases [14]. 5,10 methylene-tetrahydrofolate reductase (MTHFR) is the key rate-limiting enzyme required for the conversion of dietary folate to 5-methyltetrahydrofolate, the methyl group donor required for the remethylation of Hcy to methionine in vivo [15]. Hyperhomocysteinemia (HHcy) may be due to the presence of a thermolabile isoform of this key enzyme. A single base pair (677C > T) substitution in the human *MTHFR* gene predicts phenotypic expression of a heat-sensitive variant with reduced enzymatic activity [16]. Elevated Hcy level caused by *MTHFR* genetic variants has been demonstrated to be associated with insulin resistance [17-19]. Hcy exerts detrimental effects on a number of cell lineages including endothelial cells and neuronal cells through production of reactive oxygen species (ROS) [20]. Both acute and prolonged exposure to Hcy had detrimental effects on beta cell glucose metabolism, insulin secretory responsiveness and cell viability [20,21]. Hcy generates ROS in a redox-cycling reaction that explains the decline in viability of insulin-secreting cells, leading to reduced glucokinase phosphorylating ability, diminished insulin secretory responsiveness and cell death [22]. Based on its biological functions, this variant has been considered an ideal candidate for genetic polymorphism for predisposition to diabetes [23], as it is common in many populations studied to date and the genotype correlates highly with the plasma Hcy level in a dose-dependent fashion [24].

In recent years, there have been a number of case–control studies investigating the association between the 677 C > T polymorphism in the *MTHFR* gene and T2DM or diabetes-related complications [12,16,25-40]. However, these studies have reported conflicting results, which has prevented a definitive conclusion to date. The associations of Hcy, *MTHFR* polymorphism and T2DM are still inconclusive.

To provide an answer to these contradictory results and elucidate the possible mechanism, a meta-analysis of the published literature regarding the risk for T2DM associated with an elevated Hcy level and the *MTHFR* 677C > T was conducted. In this meta-analysis, the estimate of the genetic association of each individual study and a pooled estimate of this association were obtained. Furthermore, to test the hypothesis that elevated Hcy is causally related to increased risk of T2DM, Mendelian randomization analysis, which is an epidemiological approach based on the fact that individuals inherit genetic variants randomly from their parents, was conducted.

## Methods

### Selection of studies

All studies that investigated the association of the 677 C > T polymorphism in the *MTHFR* gene and Hcy with the development of diabetes were considered in this meta-analysis. We searched the following electronic databases from inception to December 2012: PubMed and web of science databases. As a search criterion, we used the following terms: “*MTHFR*”, “*MTHFR* 677”, “homocysteine”, “Hcy”, “diabetes”, “type 2 diabetes”, “T2DM”, and “polymorphism”.

The retrieved publications were then read in their entirety in order to assess their appropriateness for inclusion in this meta-analysis. All references cited in the studies were also reviewed to identify additional published work not indexed by PubMed and web of science databases. Case–control studies that determined the distribution of the *MTFHR* 677 C > T genotypes in diabetes and in controls free of diabetes were eligible for inclusion. Abstracts, editorials, and review articles were excluded. The search was restricted to articles in English.

### Data extraction

Data was carefully extracted from all eligible studies independently by two investigators, and an agreement was reached on all items after discussion with a third investigator. The following information was collected from each study: (1) first author's surname, publication date, subjects’ country and ethnicity; and (2) total number, definition and characteristics of cases and controls, and distribution of genotypes and alleles in all groups. For those studies that included subjects of different ethnic groups, data was extracted separately for each of the ethnic groups.

### Data analysis and statistical methods

Hardy-Weinberg equilibrium (HWE) using chi-square test was tested to in control group to determine whether it is in. Studies with controls not in HWE were subjected to a sensitivity analysis.

The meta-analysis examined the overall association of *MTHFR* 677C > T and risk of diabetes; and the contrast of homozygotes TT versus CC, the contrast TT versus (TC + CC), and the contrast (TT + TC) versus CC. All associations were indicated as odds ratios (OR) with the corresponding 95% confidence interval (CI). Then, based on the individual OR, a pooled OR was estimated. We used Stata Commands metan to estimate the mean differences for the comparisons of *MTHFR* 677 TT versus *MTHFR* 677 CC and for the comparisons of subjects with T2DM versus healthy subjects. To incorporate both within-study and between-study variability, we used random-effects model to calculate the standardized mean difference and 95% CI to pool the results for Hcy.

Heterogeneity between studies was tested using the Q-statistic, which is a weighted sum of squares of the deviations of individual study OR estimates from the overall estimate [16]. When the OR were homogeneous, Q follows a chi-squared distribution with r-1 (r is the number of studies) degrees of freedom (df). If p < 0. 05, then the heterogeneity was considered statistically significant. Heterogeneity was quantified with the I^2^ metric (I^2^ = (Q-df)/Q), which is independent of the number of studies in the meta-analysis [41]. The I^2^ takes values between 0% and 100%, with higher values denoting greater degree of heterogeneity (I^2^ = 0-25%, no heterogeneity; I^2^ = 25-50%, moderate heterogeneity; I^2^ = 50-75%, large heterogeneity; I^2^ = 75-100%, extreme heterogeneity). The pooled OR was estimated using fixed effects (Mantel-Haenszel) and random effects models. A random effect model assumes a genuine diversity in the results of various studies, and it incorporates into the calculations a between-study variance. Therefore, when there is heterogeneity between studies, then the pooled OR is estimated using the random effects model [16].

The meta-analysis consisted of the main analysis, which includes all available data; the sub-group analysis of ethnic groups. Begg’s funnel plot and Egger’s regression test (significant at p < 0.05) were used to evaluate publication bias. Extracted data was analyzed using the Stata, version 11 software (StataCorp, College Station, TX, USA).

Mendelian randomization analysis is based on the concept of Mendelian randomization, that is, the fact that one’s genes are inherited before birth by a seemingly random process analogous to treatment allocation in a randomized trial. The Mendelian randomization analysis incorporating information on both the genotype-intermediate phenotype association and genotype-disease association into one analytical framework may allow for an unbiased estimate of the intermediate phenotype-disease association. In the Mendelian randomization paradigm, an instrumental variable has to satisfy the following three criteria [42,43]: 1) the *MTHFR* 677C > T should be robustly associated with Hcy; 2) the genotype should not be associated with confounding factors that bias the association between intermediate Hcy and T2DM; 3) absence of pleiotropy, as the *MTHFR* 677C > T should exert its effect on the T2DM only through the specific intermediate Hcy. Based on available evidence, *MTHFR* 677C > T seemed to meet these assumptions well [6,39,44,45]. Thus, the Mendelian randomization coefficient estimates using *MTHFR* 677C > T as instruments would provide the causal association between Hcy and T2DM risk free of bias due to reverse causation and residual confounding. Suppose that the mutant genotype of *MTHFR* 677 C > T (TT) is associated with an increased risk of T2DM compared to the wildtype (CC) and that this effect is measured by its odd ratio (OR_TT vs CC_). Further suppose that TT is associated with a mean difference (Δ) in the level of Hcy compared with CC. OR_1_ = (OR_TT vs CC_)^1/Δ^ is an unconfounded estimate of the OR of T2DM resulting from a unit change in the Hcy. It may be more informative to rescale this OR for increments other than a unit change in Hcy. For an increment of k units the formula becomes OR_k_ = (OR_TT vs CC_)^k/Δ^[46], in this analysis, we used 5 μmol/l increase in serum Hcy to calculate the OR [47].

## Results

### Summary statistics

**Figure 1:**
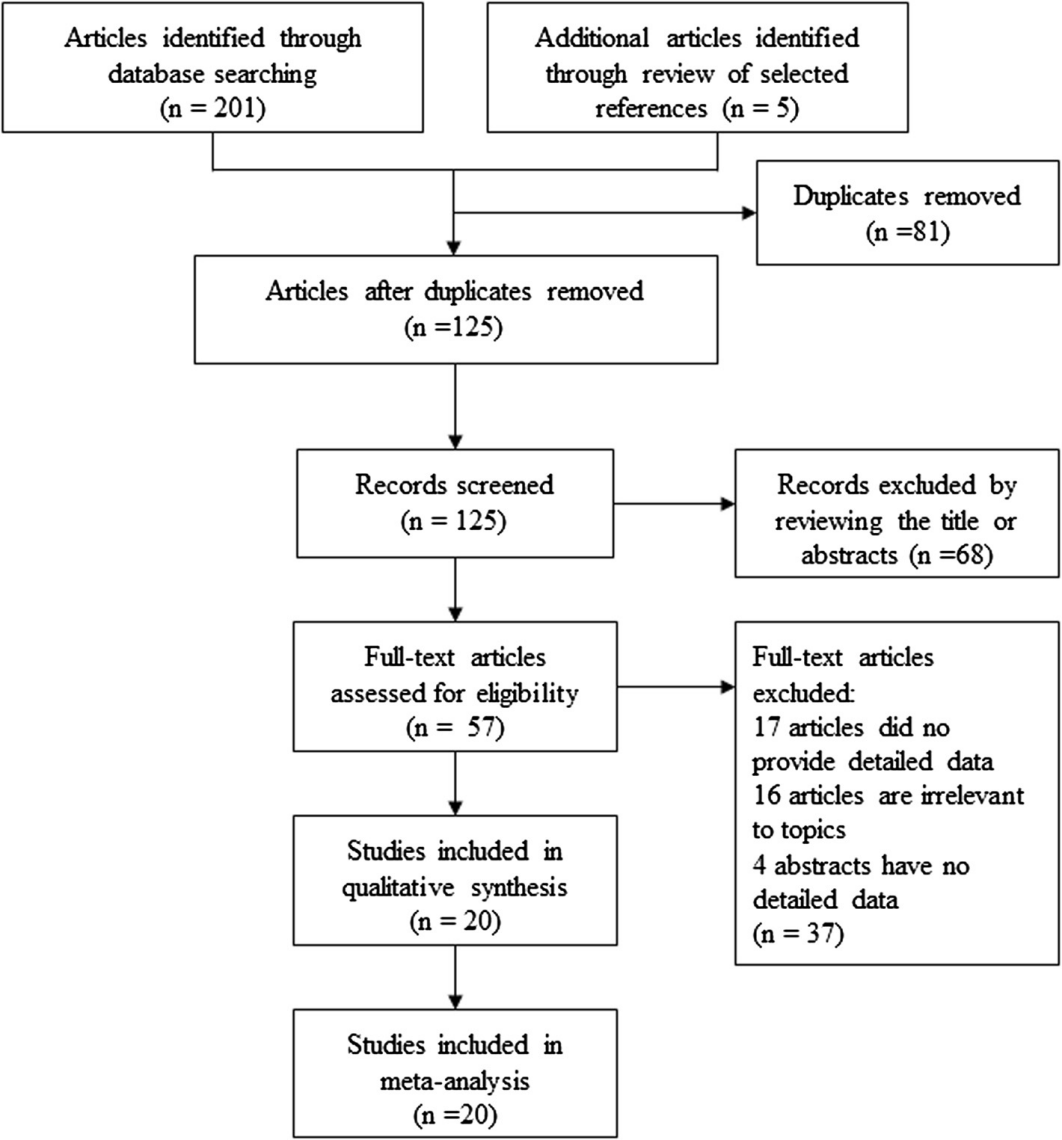
PRISMA flow diagram for selection of studies in the meta-analysis.

The seventeen studies provided 4011 cases and 4303 controls [12,25-40] (Figure 1). The allele C was the most common. The frequencies of the CC genotype were the highest in controls and in cases, while that for genotype TT was the lowest. Three studies only reported the association between blood Hcy level and *MTHFR* 677 C > T in diabetes patients [48-50]. In three studies [34-36], the distribution of the genotypes in the control group was not in HWE (p < 0.05) (Table 1).

**Table 1:** **The distribution of the *MTHFR *** **677 C > T genotype for case and control and the allele frequencies**

**First author**	**Year**	**Ethnical decent**	**Distribution of *MTHFR *** **genotypes (n(%))**						**Frequency of *MTHFR *** **alleles (%)**				**P-valueHWE**
			**CC**		**CT**		**TT**		**C**		**T**		
			**Case**	**Control**	**Case**	**Control**	**Case**	**Control**	**Case**	**Control**	**Case**	**Control**	
Ndrepepa et al. [25]	2008	Germany	225(44)	690(43)	215(42)	740(46)	67(13)	184(11)	/	/	/	/	0.49
Mazza et al. [12]	2005	Italy	35(33)	35(29)	47(45)	66(55)	23(22)	19(16)	/	/	/	/	0.19
Bluthner et al. [26]	1999	Poland	137(47)	67(45)	115(39)	68(45)	41(13)	15(10)	/	67	/	33	0.71
Benes et al. [27]	2001	Czech	166(48)	86(41)	152(43)	106(51)	31(8)	17(8)	69	67	31	33	0.05
Yilmaz et al. [28]	2004	Turkish	121(49)	101(47)	98(39)	93(44)	30(12)	20(9)	68	69	32	31	0.83
Eroglu et al. [29]	2007	Turkish	51(50)	63(49)	45(44)	58(45)	7(7)	7(6)	71	71	29	29	0.17
Tutuncu et al. [30]	2005	Turkey	41(45)	52(52)	39(43)	43(43)	11(12)	5(5)	60	70	40	30	0.29
Soares et al. [31]	2007	UK	10(55)	9(56)	6(33)	5(31)	2(11)	2(13)	/	/	/	/	0.36
Angeline et al. [32]	2009	Indian	88(73)	80(80)	32(27)	20(20)	0	0	88	90	12	10	0.57
Mehri et al. [33]	2010	Tunisian	50(43)	66(57)	49(43)	38(33)	16(14)	12(10)	65	73	35	27	0.07
Sun et al. [34]	2004	Chinese	102(46)	74(57)	76(35)	34(26)	42(19)	22(17)	64	70	56	30	0.001
Mtiraoui et al. [35]	2007	Tunisia	163(45)	270(68)	135(38)	94(24)	62(17)	36(9)	64	79	36	21	0
Sun et al. [36]	2003	Chinese	84(40)	31(54)	75(36)	16(28)	49(24)	10(18)	58	68	42	32	0.01
Movva, et al. [37]	2011	Indian	68(68)	91(91)	32(32)	9(9)	0	0	84	95	16	4	0.05
Tavakkoly Bazzaz et al. [38]	2010	Iran	148(53)	113(55)	102(36)	80(39)	31(11)	14(7)	71	76	26	24	0.21
Benrahma et al. [39]	2012	Morocco	160(57)	114(44)	97(35)	122(47)	25(9)	26(10)	74	67	26	33	0.19
Sharaf et al. [40]	2012	Egypt	24(48)	13(65)	17(34)	5(25)	9(18)	2(10)	65	78	35	22	0.13

The significance level (p-value) for Hardy-Weinberg equilibrium (HWE) testing for controls is shown.

### Pooled difference in mean Hcy level between MTHFR genotypes in type 2 diabetes patients

**Figure 2:**
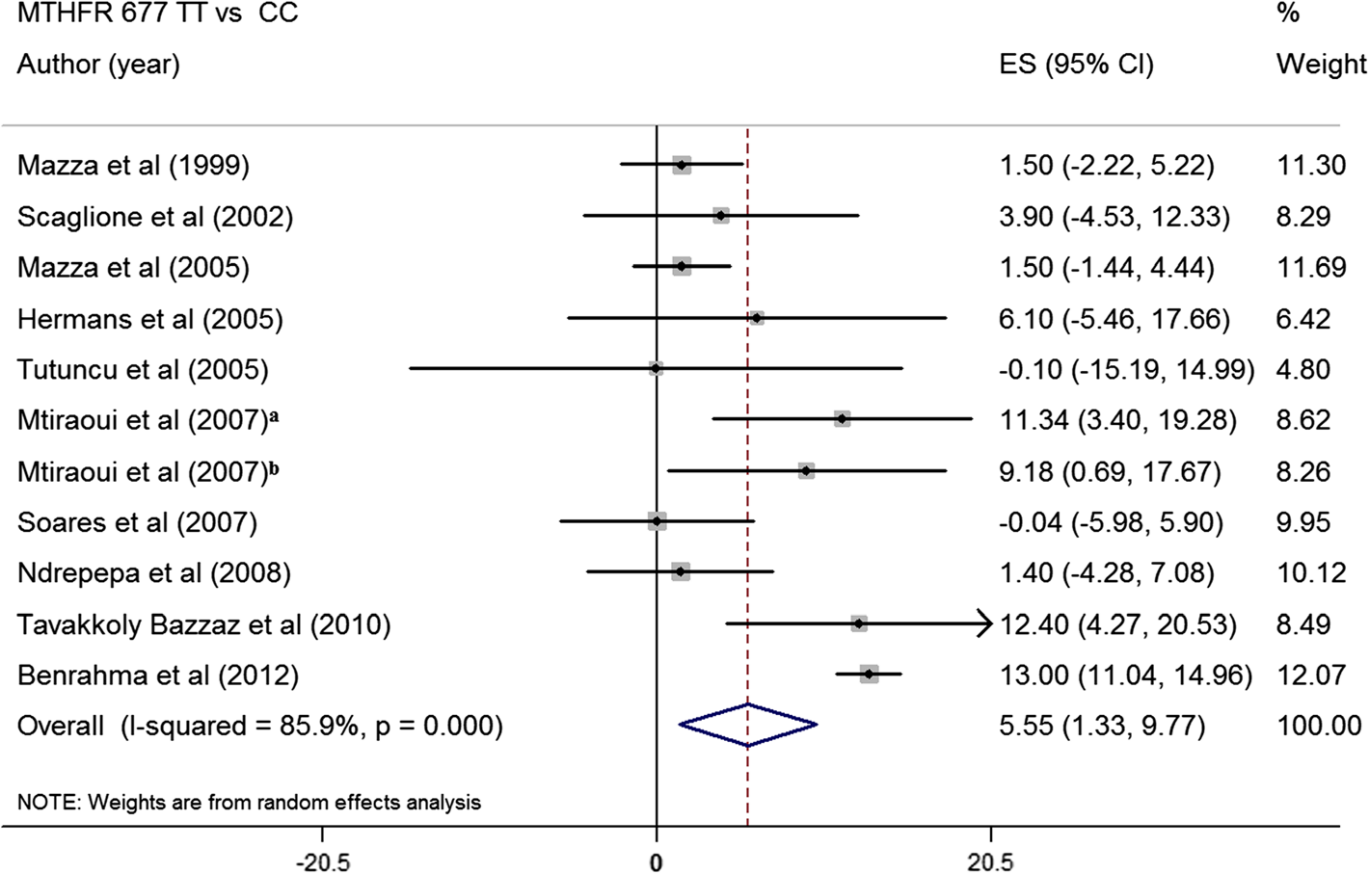
**Summary estimates for the effect size (ES) in plasma Hcy between the *MTHFR *genotypes (TT vs CC) in type 2 diabetes patients. **^a^T2DM patients with nephropathy, ^b^T2DM patients without nephropathy.

**Figure 3:**
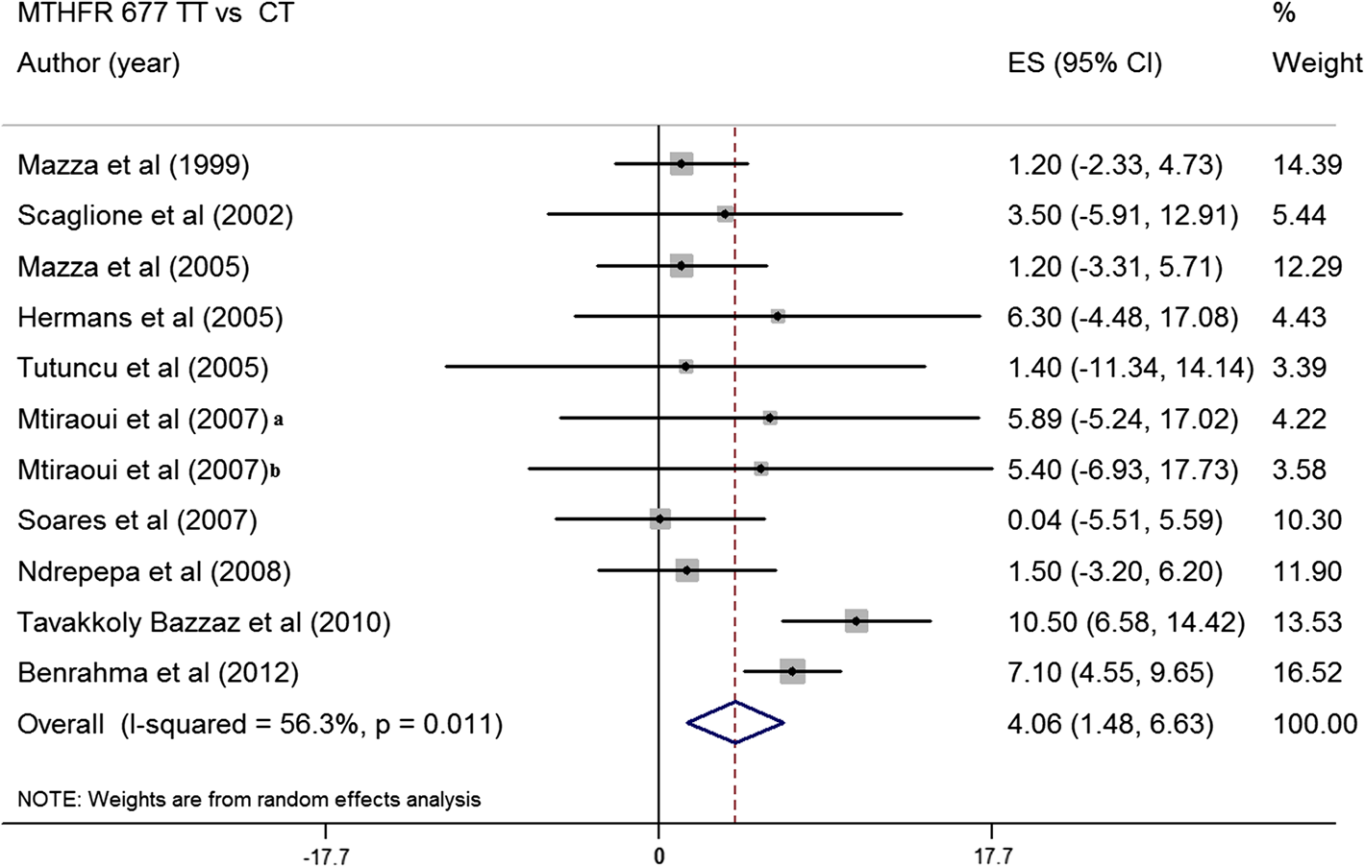
**Summary estimates for the effect size (ES) in plasma Hcy between the *MTHFR *genotypes (TT vs CT) in type 2 diabetes patients. **^a^T2DM patients with nephropathy, ^b^T2DM patients without nephropathy.

Eleven studies (2498 T2DM) [12,25,30,31,35,38,39,48-50] were identified that satisfied inclusion criteria and expressed the between-group difference in plasma Hcy level in terms of the arithmetic mean and standard deviation. In all these studies, the mean Hcy concentration was greater in subjects with *MTHFR* 677TT than in those with the other two genotypes. The absolute pooled mean Hcy concentration in subjects with *MTHFR* 677TT was 5.55 μmol/L (95% CI, 1.33 to 9.77) greater than that in subjects with *MTHFR* 677CC (p = 0.012) (Figure 2). While, the subjects with *MTHFR* 677TT had 4.06 μmol/L higher Hcy concentration than subjects with *MTHFR* 677CT (p = 0.008) (Figure 3). The sensitivity analysis regarding the *MTHFR* 677C > T not being in HWE did not significant change the results.

### The association between MTHFR 677 C > T and risk of type 2 diabetes

**Figure 4:**
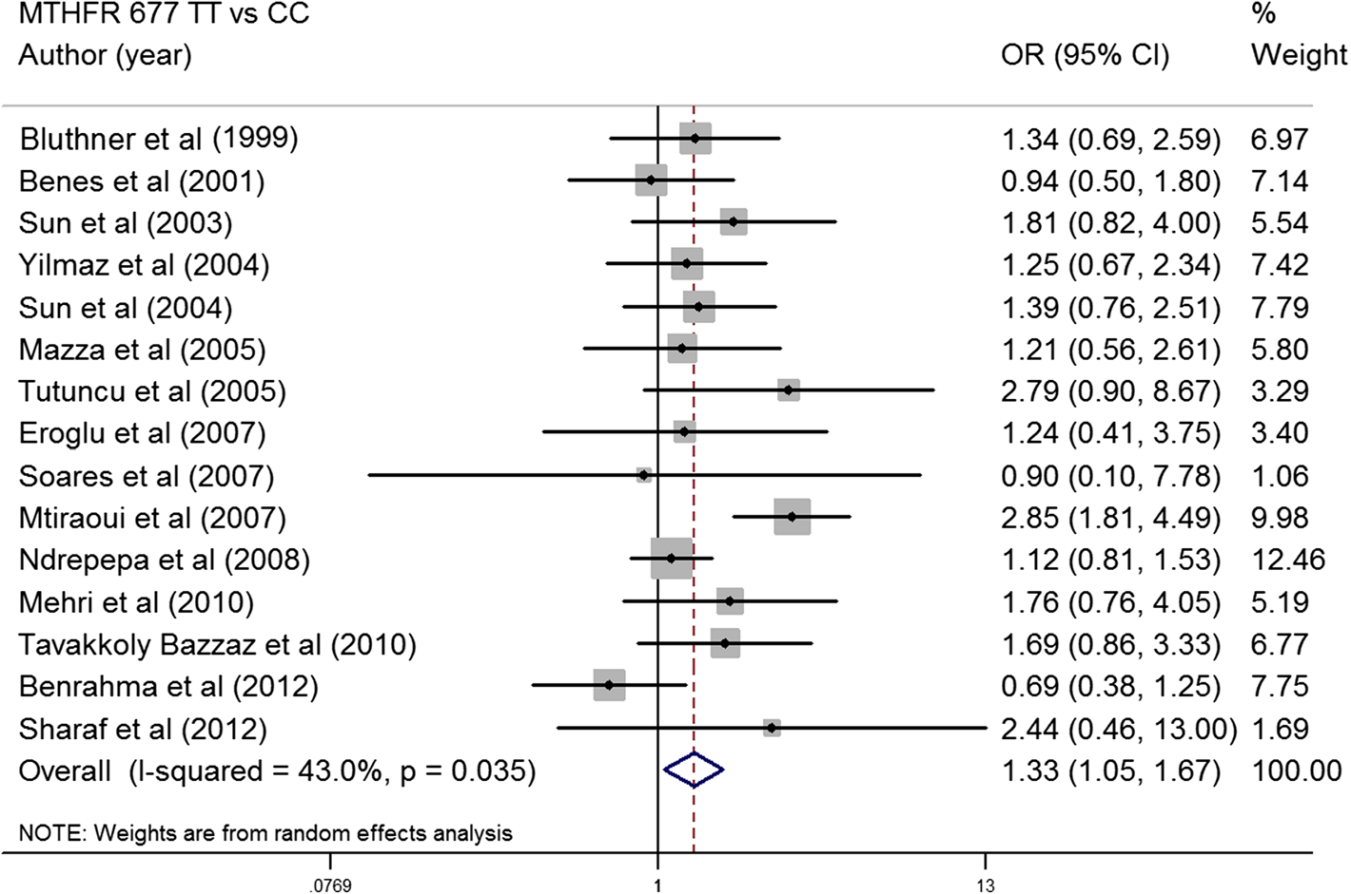
**Summary estimates for the OR of *MTHFR *677 C > T in genotype contrasts (TT vs CC), the OR with the 95% confidence interval is shown.** Studies are displayed by descending order of publication year.

**Figure 5:**
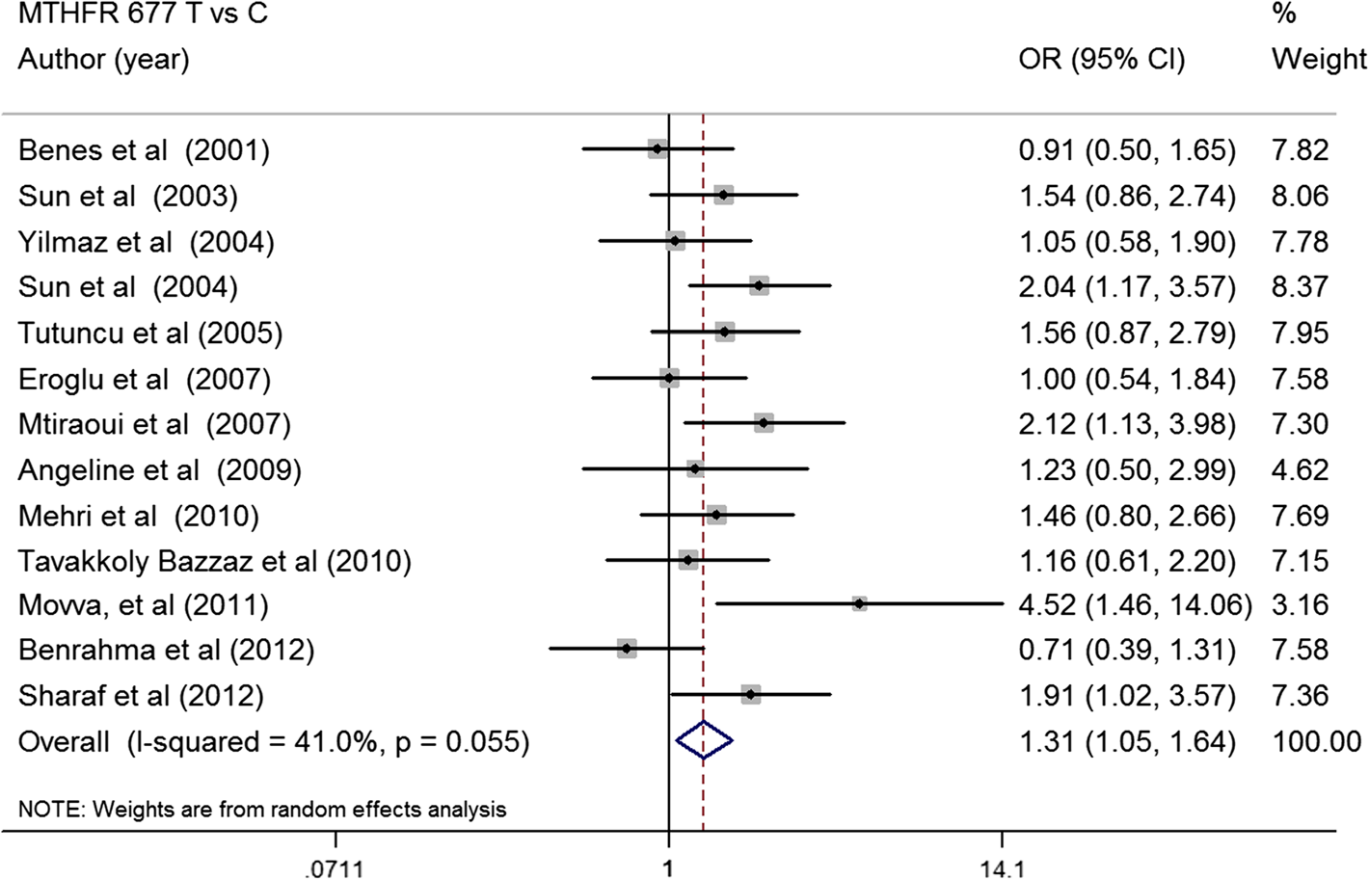
**Summary estimates for the OR of *MTHFR *677 C > T in allele contrasts (T vs C), the OR with the 95% confidence interval is shown.** Studies are displayed by descending order of publication year.

The main analysis for investigating the association between *MTHFR* 677TT and risk of developing T2DM relative to the 677CC revealed significant heterogeneity (p for heterogeneity = 0.035, I^2^ = 43%) between 17 studies; the random effect (RE) pooled OR was significant: RE OR = 1.33 (1.05-1.67) (p = 0.021) (Figure 4). Overall, the T allele of the *MTHFR* 677 C > T conferred a greater risk for T2DM [RE OR = 1.31(1.05-1.64) (p = 0.032), I^2^ = 41.0%, p for heterogeneity =0.055] (Figure 5). The random-effect pooled OR of the association for *MTHFR* 677 TT vs CT was 1.29 (95% CI, 1.09-1.51) (p = 0.021). *MTHFR* 677 TT showed a significantly higher risk for T2DM compared with *MTHFR* 677 CC + CT [Fixed effect (FE) OR = 1.32(1.14-1.54) (p = 0.034), I^2^ = 0.0%, p for heterogeneity =0.686]. *MTHFR* 677 TT + CT also showed a significantly higher risk for T2DM compared with *MTHFR* 677 CC [RE OR = 1.12 (1.07-1.34) (p = 0.029), I^2^ = 42.0%, p for heterogeneity =0.046]. Subgroup analysis was also performed to ensure that the application of the method of estimating the causal effect is unbiased. Subgroup analysis was grouped by studies that reported results on both associations (*MTHFR* 677 C > T-T2DM effects and *MTHFR* 677 C > T-Hcy effects) and by those reported only a single association result. The results showed similar significant effect sizes between both groups (both: 1.44 (0.93-2.23), Single: 1.35 (1.03-1.75).

### The associations between plasma Hcy and type 2 diabetes

**Figure 6:**
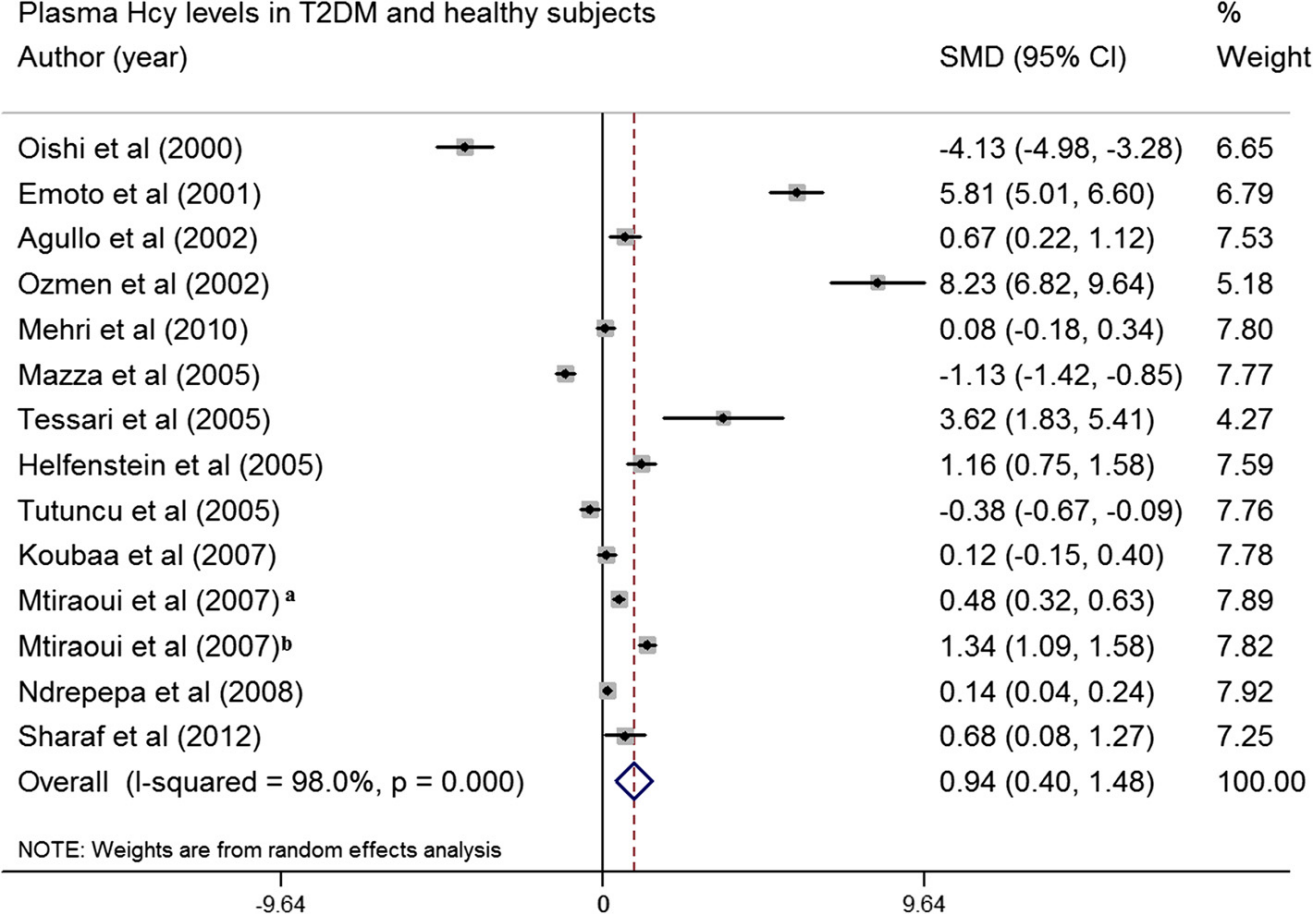
**Forest plot of standardized mean difference (SMD) in Hcy between subjects with and without type 2 diabetes in included studies. **^a^T2DM patients with nephropathy, ^b^T2DM patients without nephropathy.

Figure 6 showed a forest plot of standardized mean difference (SMD) in Hcy between subjects with and without T2DM in included studies. The main analysis for this association revealed significant heterogeneity (p for heterogeneity < 0.001, I^2^ = 98.0%) between the studies. In 14 of these studies [7,8,12,13,25,30,33,35],[40,51-54], the mean Hcy concentration was greater in patients with T2DM than in control subjects (Figure 6). The absolute pooled mean Hcy concentration in patients with T2DM was 0.94 μmol/L (95% CI, 0.40-1.48) greater than that in control subjects in random model (p = 0.017). We further conducted a subgroup analysis of ethnic groups, all results from Europe, Asia and Africa showed significant differences in Hcy levels between subjects with or without T2DM. Additionally, subgroup analysis was also grouped by studies that reported results on both associations (*MTHFR* 677 C > T-T2DM effects and *MTHFR* 677 C > T-Hcy effects) and by those reported only a single association result. The results showed similar significant effect sizes between both groups (both: 5.94 (0.30-11.59), Single: 3.03 (−0.02-6.08). In addition, Begg’s and Egger’s tests did not show evidence for the presence of substantial publication bias for the Hcy-T2DM association in different inheritance models (data not shown).

### Causal association of Hcy with risk of type 2 diabetes by Mendelian randomization

**Figure 7:**
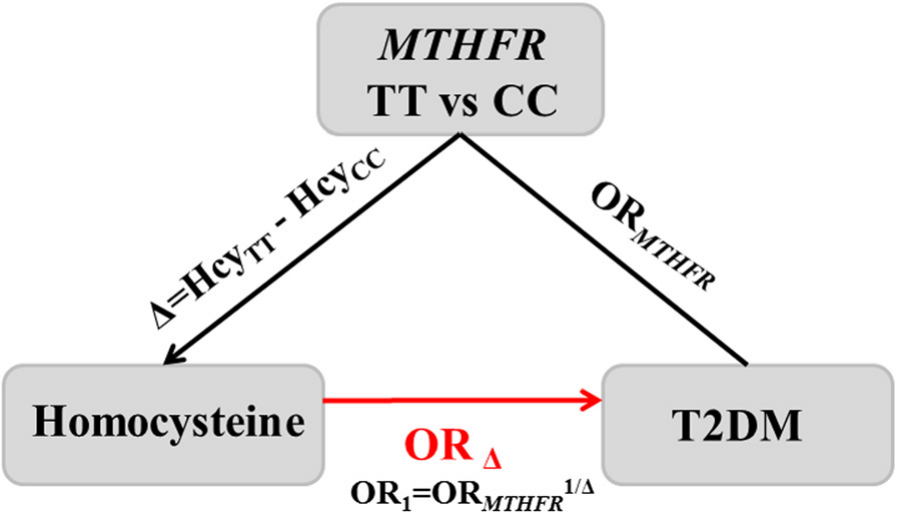
**The potential causal relation between Hcy and T2DM risk was explored by using Mendelian randomization.** Calculation of an unconfounded estimate of the effect of an increase in Hcy of 1 μmol/L on the risk of T2DM based on the concept of Mendelian randomization.

Figure 7 showed the predicted OR of T2DM associated with per unit increase in direct or indirect measures of Hcy using *MTHFR* 677 C > T as an instrumental variable for Hcy. Increased Hcy levels were strongly associated with increased risk of type 2 diabetes. The estimated causal OR for 5 μmol/L Hcy increment was 1.29 (1.04-1.59).

## Discussions

Overall, we found that *MTHFR* 677 T was significantly associated with elevated plasma Hcy concentration. The pooled mean Hcy concentration was greater in subjects with T2DM than in those without T2DM. Our findings from Mendelian randomization approach supported the hypothesis that elevated Hcy is causally related to increased risk of T2DM.

Previous studies have given conflicting results regarding the circulating Hcy levels in patients with diabetes. An association between Hcy level and diabetic complications was seen in type I diabetes, but not among T2DM [7]. Very recently, a study on 105 patients with T2DM and normal renal function also found that in patients with T2DM, basal level of Hcy was 35% lower compared with healthy subjects [12]. The reduced Hcy level may be explained by an influence of hyperglycemia on acceleration in the hepatic transsulfuration pathway due to insulin disorder [12], subsequently, elevated glucocorticoids decreased Hcy level [55]. Cell culture and animal studies also showed that the diabetic status may reduce rather than increase the circulating Hcy level due to enhanced Hcy catabolism [55,56]. However, it should be emphasized that this data was derived from animal models of experimental diabetes and cannot provide solid evidence that a similar relationship between diabetic status and Hcy level exists in patients with diabetes. Moreover, many other studies found that plasma Hcy levels were significantly higher in diabetic patients than in subjects without diabetes. In the present meta-analysis, the main analysis for weighted mean difference in Hcy between subjects with and without T2DM in all included studies revealed significant heterogeneity between the studies. Therefore, we used random model to estimate the pooled mean Hcy. Similarly, the absolute pooled mean Hcy concentration in individuals with T2DM was also significantly higher than that in control subjects in random model. We speculated that a large part of the discrepancy resulted from the heterogeneity of patients included in these studies with regard to the stage of disease, status of renal function or presence of macroangiopathy [4,9]. Furthermore, the opposing influences on circulating Hcy levels exercised by the diabetic status per se and by a number of risk factors that relate to or are accentuated by diabetes may also help explain the controversy [25].

Importantly, the heterogeneity may also result from the genetic background of the patients. A common variant (*MTHFR* 677 C > T) of the enzyme MTHFR involved in the Hcy metabolic pathway may affect Hcy concentration [57]. Among the diabetic group, patients carrying the T allele showed a increased Hcy level in comparison to those having the native genotype CC [23]. This contradicted a previous report which suggested that double heterozygosity was associated with reduced enzyme activity and high Hcy level [58]. Interestingly, the *MTHFR* 677 C > T appears to have a sex-specific effect on Hcy, Hcy level was slightly higher in men than in women [59]. Men carrying TT genotype appear to be at higher risk of HHcy than women with this genotype [60]. Plasma estrogen was found to decrease in *MTHFR* CC, but not in TT homozygous postmenopausal women [61]. The precise mechanism by which estrogen could affect Hcy metabolism is not known. But the sex-specific effect may contribute to the previous contradicted results. Our meta-analysis gave strong evidence that the *MTHFR* was associated with plasma Hcy in patients with T2DM. *MTHFR* 677TT have significantly higher plasma Hcy than other genotypes, which are consistent with previous results in diabetic subjects [25].

Because elevated plasma Hcy level in patients with T2DM has been reported, it may be readily postulated that the 677 C > T *MTHFR* gene polymorphism might be involved in the development of T2DM [23]. However, no literature data directly associates the *MTHFR*-linked Hcy metabolism with T2DM. To date, the relationship between the *MTHFR* 677 C > T polymorphism and T2DM is less clear. Previous studies found an association between mutant homozygous genotype for *MTHFR* 677 C > T and diabetic retinopathy in individuals with T2DM [62]. Similarly, another study has also found that the *MTHFR* 677 C > T predisposes T2DM patients to the development of diabetic retinopathy [63]. One study suggested the *MTHFR* mutant genotype as a possible risk factor for the development of left ventricular hypertrophy in T2DM [28]. The TT genotype was more frequent in T2DM patients than in healthy controls (13.9% vs. 10.3%). Of interest is that the Brazilian population holds the lowest frequency ever reported for the TT genotype (9%) [53], and the highest (19.1%) is in Chinese population [34]. In fact, the association between the *MTHFR* 677 C > T and diabetes was not found in some studies including Brazil [53], China [34], Germany [25]. The reasons why there are conflicting results in studies concerning *MTHFR*677 C > T and T2DM are still not understood, however, an important reason for conflicting results may relate to the different genetic background of patients included in the studies. The frequency of *MTHFR* 677TT varies in different ethnic groups, for example, the 677TT genotype frequency is the lowest in Moroccan patients and controls (8.87% and 9.92%, respectively) [39]. Similarly, low frequency of 677TT (9%) was also reported in a Brazilian population [53]. In contrast, a high frequency (19.1%) of the 677TT genotype was reported in a Chinese population [34]. Furthermore, small sample size, study design flaws or other biases may be more common reasons than true racial heterogeneity for the observed discrepancies between studies addressing genetic risks [64,65]. The meta-analysis presented here included data from case–control association studies that investigated the relationship between *MTHFR* polymorphism and T2DM. The strength of the present analysis, however, is based on the aggregation of published case–control studies. The overall results indicated an association of *MTHFR* 677 C > T with T2DM and *MTHFR* TT genotype increases the risk of T2DM.

Our study is the first meta-analysis to date which has pooled all the data available to investigate the associations of *MTHFR*-linked Hcy and T2DM. Therefore we have provided the most extensive data on this issue. As with all meta-analyses, some limitations are also presented here. First, the lack of clinical homogeneity between the subjects in the included studies, although our inclusion criteria ensured that the selected studies were broadly similar, it was not possible to eliminate all sources of heterogeneity. Second, we pooled the data from different ethnicities together, genetic heterogeneity among diverse ethnic populations leads to some unavoidable bias. Third, many studies did not include information regarding vitamin status at the time of Hcy measurement, which prevented examination of the interaction between nutritional status, Hcy, genotypes, and diabetic risk. Differences in sampling protocols and methods of Hcy measurement may have contributed to variation between studies. Hcy measurement using different high-performance liquid chromatography methods has been reported to vary by 6 to 7% among different laboratories [66]. Fourthly, exclusion of studies which didn’t provide adequate information might contribute to the publication bias. We also cannot exclude the possibility of bias related to the exclusion of non-English language publications, although Begg’s and Egger’s tests did not show evidence for the presence of substantial publication bias with respect to the small effect sizes. Fifthly, although we observed elevated Hcy is causally related to increased risk of T2DM, recent clinical trial showed that lowering Hcy levels by daily supplementation with folic acid and vitamins B_6_ and B_12_ did not reduce the risk of developing T2D among women at high risk for CVD [67]. We specialated that the associations of Hcy with T2D may be modified by environmental factors such as dietary folate, as well as the potential gene-environment interactions involved in the development of T2D. Finally, the association of the *MTHFR* variant with T2D was not observed in large-scale GWAS studies with huge sample sizes. The possible reason is that this association is likely to be due to the combined effect of genes, environmental factors, and their interactions. However, most investigators conducting GWAS do not consider gene-environment interactions in their search for new genes due to a current lack of efficient statistical methods for detecting interactions in high-volume genetic data.

## Conclusions

In summary, our results provided evidence that the TT genotype of *MTHFR* C677T contributes to susceptibility to T2DM, and supported the hypothesis that elevated Hcy is causally related to increased risk of T2DM. The existence of gene-environment interactions may explain the discrepancy of results obtained in individual genetic association studies. Therefore, case–control studies that investigate gene-environment interactions might help further elucidate the genetics of T2DM.

## Consent

Written informed consent was obtained from the patient for the publication of this report and any accompanying images.

## Competing interests

The authors declare that they have no competing interests.

## Authors’ contributions

TH, JR, and DL designed research; TH, JR analyzed data; TH, LR, JH wrote the paper. DL had primary responsibility for final content. All authors read and approved the final manuscript.
